# Perspectives on Point-of-Care Ultrasonography Credentialing and Privileging: A Qualitative Study

**DOI:** 10.1001/jamanetworkopen.2025.38759

**Published:** 2025-10-22

**Authors:** Stephanie M. Conner, James E. Anstey, Meghan K. Thomas, Patricia A. Carney, Maya D. Fiore, Carley E. Little, Trevor P. Jensen, Kevin M. Piro

**Affiliations:** 1 Division of General Medicine, Department of Medicine, Columbia University Irving Medical Center, New York, New York; 2 Division of Hospital Medicine, Department of Medicine, Oregon Health & Science University, Portland; 3 Division of Hospital Medicine, Department of Medicine, Medical University of South Carolina, Charleston; 4 School of Medicine, University of South Carolina–Greenville; 5 Division of Hospital Medicine, Department of Medicine, University of California San Francisco

## Abstract

**Importance:**

Point-of-care ultrasonography (POCUS) use is rapidly expanding across several acute care specialties, yet no defined standards currently exist to guide health systems in regulation of POCUS use or clinician best practices.

**Objective:**

To describe existing POCUS credentialing and privileging policy design and implementation in a purposive sample of health care institutions across the US.

**Design, Setting, and Participants:**

This qualitative study used a cross-sectional, mixed-methods analysis of POCUS policies and procedures conducted between July 23 and October 28, 2024, and was guided by the Practical, Robust Implementation and Sustainability Model (PRISM) framework. Qualitative analyses used an interpretive phenomenological approach. Participants were POCUS leaders at health care institutions with existing POCUS policies, representing diverse geographic areas, sizes, and practice settings.

**Exposures:**

Thirty-item online survey and 60-minute key informant structured interviews.

**Main Outcomes and Measures:**

Quantitative survey findings and qualitative analyses of current POCUS policies and practice patterns related to content, implementation, adoption, facilitators, and barriers to use.

**Results:**

Twenty survey and interview participants were included (mean [SD] age, 41.17 [3.79] years; 15 [75.0%] male) and 19 POCUS policies were analyzed. Interviewees reported that successful policy development was influenced by contextual factors (eg, institutional motivators, external forces), policy infrastructure, diverse stakeholder engagement, ability to anticipate and address multilevel barriers, and strategies to maximize policy reach, adoption, and sustainability. Key insights from interviewees highlighted the importance of multidisciplinary POCUS committees and purposeful policy design tailored to align with institutional POCUS practice patterns and values. Leaders stressed the need for careful consideration of policy outcomes and institutional capabilities prior to implementation. While features of policies varied, most delineated specific POCUS applications within the policy and all required institutional training or proctoring from institutional POCUS champions.

**Conclusions and Relevance:**

This study identified common themes and barriers in POCUS implementation and strategies used by exemplar institutions to aid other programs in addressing POCUS privileging and establishing standards for safe and effective POCUS use in patient care. The findings suggest POCUS policy development is important for standardizing its use within institutions, guiding training requirements, and establishing oversight procedures.

## Key Points

**Question:**

What factors play a role in point-of-care ultrasonography (POCUS) privileging and credentialing policy implementation at health care institutions across the US?

**Findings:**

In this qualitative study, 20 POCUS leaders reported that successful POCUS policy implementation was driven by institutional and external contextual factors, addressing multilevel barriers, policy-specific characteristics, and institutional POCUS capabilities. Policy specifications varied across institutions, though all required local expertise to implement and sustain; the most common theme was establishing diverse stakeholder engagement during policy development and implementation.

**Meaning:**

The findings suggest POCUS policy implementation guides safer POCUS-informed care and is more likely to succeed when barriers, contextual determinants, and implementation infrastructure supporting policy development are thoughtfully considered.

## Teaser

This qualitative study evaluates credentialing and privileging policies for diagnostic point-of-care ultrasonography (POCUS) implementation in US health care institutions based on interviews with POCUS leaders.

## Introduction

Over the past decade, diagnostic point-of-care ultrasonography (POCUS) use has rapidly expanded across several acute care specialties.^1,2^ This technology is improving diagnosis, prognostication, and patient experience in the emergency department, critical care, and inpatient acute care environments.^3,4,5,6,7,8,9,10,11,12,13^ Despite enthusiasm for POCUS, adapting it to clinical practice has experienced growing pains. Some medical specialties, such as emergency medicine (EM), have established competencies and recommendations to oversee safe and effective implementation in patient care. However, many emerging POCUS programs, particularly within primary care specialties (eg, internal medicine, family medicine, and pediatrics), have developed training programs while struggling to build needed safeguards such as quality assurance (QA) of images, appropriate educational resources, and privileging and credentialing policies.^14,15^

Hospitals outline clinicians’ scope of practice through credentialing and privileging policies, as required by the Centers for Medicare & Medicaid Services.^16^ Credentialing involves reviewing clinicians’ general qualifications, while privileging assesses their competencies to grant specific practice rights within an institution. Both processes are governed by legal, regulatory, and accreditation standards, with regular reevaluations to maintain quality. While to our knowledge no direct empirical evidence exists proving that privileging and credentialing policies improve patient outcomes or lead to safer patient care, they have been universally considered foundational for patient safety since the 1950s.^17^ Additionally, many national societies have recommended institutional development of POCUS policies.^18,19,20,21,22^ Although need for POCUS privileging remains debated, efforts to standardize processes are underway, as current practices vary widely and no universal approach exists.

In this study, we surveyed and conducted key informant interviews with POCUS leaders to identify and describe processes associated with the development and implementation of institutional diagnostic POCUS credentialing and privileging policies (hereafter, “policies”). This work was guided by the Practical, Robust Implementation and Sustainability Model (PRISM) framework, which considers how a given program or intervention design, contextual elements (external environment, participants), and implementation and sustainability infrastructure influence outcomes.^23^

## Methods

### Design

This qualitative study used a cross-sectional mixed-methods approach. The quantitative component involved a 20-item survey; the qualitative component included a 60-minute structured interview with the same POCUS leaders who completed the survey. The study received institutional review board approval from all participating institutions. Oral informed consent was obtained from participants completing both the survey and the interview. Participants were not compensated. The study followed the Standards for Reporting Qualitative Research (SRQR) reporting guideline.

### Participants

A purposive sample of POCUS leaders was identified using a consensus approach among the author group. Inclusion criteria included having either an established or in-progress POCUS policy within the host institution. Then, the study team met to identify and discuss potential participants based on certain characteristics relevant to the study—such as geographic region, practice setting, and age—and who were perceived as likely to provide a broad set of opinions.

### Instrument Development, Testing, and Data Collection

A study-specific survey was developed to collect demographic and clinical information (n = 12 items), modeled after common questions in survey instruments, as well as several variables related to policy status (n = 18 items) guided by the PRISM framework (eAppendix 1 in Supplement 1). Prior to administration, the survey was pilot tested by a study author (P.A.C.) using cognitive interview techniques.^24^ Revisions and retesting were repeated until no additional changes were needed. The survey was administered between July 23 and October 28, 2024, via an online survey platform and was distributed to participants via electronic mail. Additionally, participants were asked to upload their POCUS policy for analysis. All surveys were completed before key informant interviews and were deidentified prior to analyses.

A 17-question interview guide was developed for key informant interviews, guided by the PRISM framework, and focused on policy development and implementation (eAppendix 2 in Supplement 1). Survey responses and the uploaded policies were reviewed in advance to prepare interview probes. Interviews were both arranged and conducted by physician coauthors with extensive POCUS expertise (S.M.C., J.E.A., M.K.T., T.P.J., and K.M.P.). Interviews occurred at times convenient for both participants and interviewees between July and October 2024, and they lasted approximately 60 minutes. Interviews were recorded and transcribed verbatim for qualitative analyses, and the interview guide did not change over the course of the study. Qualitative data were deidentified prior to analyses.

### Statistical Analysis

All study data were maintained on institutionally approved password-protected computers. Frequencies were determined to check for outliers and extent of data missingness. Descriptive statistics, including frequencies, percentages, means, SDs, and ranges, were used to analyze survey data. Analyses were performed using IBM SPSS, version 29 (IBM Corp). We used an interpretive phenomenological approach^25^ for qualitative analyses, specifically because it is designed to explore and describe participants’ lived experiences—in this case, with developing and implementing POCUS policies. A strength of interpretive phenomenology is that it produces an account of lived experience using its own terms rather than an account prescribed by preexisting preconceptions. It is particularly useful when examining complex and ambiguous topics. This approach is also flexible, which helped to discern uncertain and complex multidimensional human and social constructs. We applied the PRISM framework to our qualitative findings to understand the context of POCUS policy development, implementation strategies, and outcomes.

Using a descriptive approach, 3 authors (S.M.C., P.A.C., and K.M.P.) independently and iteratively reviewed interview transcripts to identify emergent themes and how they clustered into meaningful groups. We used member checking and bracketing techniques to put aside prejudgments and identify potential biases that may have emerged, and we held weekly consensus meetings to compare and finalize emergent themes, definitions, and alignment with the PRISM framework. Exemplar quotations that best reflected the themes and their definitions were selected to illustrate findings. As a final validation step, we shared findings with a selected group of study participants, which resulted in minor changes to the thematic definitions and PRISM framework components. A study author (S.M.C.) then used classic content analysis to analyze the components of available POCUS policies.^26^

The reflexology statement on the expertise and characteristics of the individuals involved in the qualitative analyses includes that authors S.M.C. and K.M.P. are both midcareer White physicians, one cisgender female and one cisgender male, who are content and context experts. Author P.A.C. is a late-career, White, nonphysician, cisgender female, doctorally trained and experienced mixed-study methodologist who served as the qualitative research expert, questioned assumptions, and fostered self-reflective strategies to mitigate potential biases during the analysis process.

## Results

Our quantitative and qualitative analysis included 20 survey and interview participants with a mean (SD) age of 41.17 (3.79) years (range, 36-48 years) (Table 1). Fifteen participants (75.0%) were men and 5 (25.0%) were women.

**Table 1.** Demographic and Clinical Practice Characteristics of Participants

Characteristic	Participants (N = 20)^a^
Age, mean (SD) [range], y	41.2 (3.8) [36-48]
Missing	2
Gender identity	
Man	15 (75.0)
Woman	5 (25.0)
Time since completion of training, mean (SD), y	11 (4.5)
Range according to year	1999-2019
Hospital medicine as primary specialty	20 (100)
Has formal POCUS role at institution^b^	20 (100)
Support for POCUS role, FTE	
None	3 (15.0)
0-0.2	13 (65.0)
0.21-0.4	2 (10.0)
0.41-0.6	2 (10.0)
Duration of POCUS use in clinical care, mean (SD) [range], y	7.2 (2.3) [3-10]
Practice setting	
Tertiary or quaternary referral center	15 (75.0)
Community medical center	3 (15.0)
Public or safety-net center	2 (10.0)
Hospital size	
Small (<100 beds)	0
Medium (100-499 beds)	3 (15.0)
Large (≥500 beds)	17 (85.0)
Practice location	
Rural	0
Suburban	2 (10.0)
Urban	18 (90.0)
Hospital affiliated with university	
Yes	18 (90.0)
No	2 (10.0)
Hospital has an IM residency training program	20 (100)
Practice region	
West	7 (35.0)
Midwest	5 (25.0)
South	5 (25.0)
Northeast	4 (20.0)

Abbreviations: FTE, full-time equivalent; IM, internal medicine; POCUS, point-of-care ultrasonography.

^a^ Data are presented as number (percentage) of participants unless otherwise indicated.

^b^ For example, director of POCUS education or POCUS director.

### Quantitative Findings

Of the survey respondents, 14 (70.0%) indicated their institution had finalized and implemented POCUS policies that were either intermittently or broadly used (Table 2). However, only 7 (35.0%) stated that more than 75% of diagnostic POCUS examinations being used at their institution for clinical practice (noneducational) were being performed by privileged clinicians, suggesting privileges were not closely associated with POCUS use.

**Table 2.** Status of POCUS Policies at Participants’ Institutions

Response variable	Participants, No./total No. (%)
Current status of POCUS credentialing and privileging policies	
Started but not finalized	3/20 (15.0)
Anticipated within 9 mo	1/20 (33.3)
Anticipated within >9 mo	2/20 (66.7)
Finalized but not implemented	3/20 (15.0)
Finalized and intermittently used	7/20 (35.0)
Finalized and broadly used	7/20 (35.0)
If policy exists, external factors that helped shape it (ranked first)^a^	
Patient safety and quality care	7/17 (41.2)
Medicolegal culture and/or concerns	5/17 (29.4)
Reimbursement	3/17 (17.6)
Evidence-based medicine practices	1/17 (5.9)
Other	1/17 (5.9)
Physician specialties most likely to seek POCUS credentialing	
Emergency medicine attending physicians	13/17 (76.5)
Hospitalist attending physicians	3/17 (17.6)
OBGYN attending physicians	1/17 (5.9)
Estimated proportion of POCUS examinations done by nonprivileged vs privileged clinicians	
Most (>75%) by nonprivileged clinicians	3/17 (17.6)
Approximately 25%-75% by nonprivileged clinicians	5/17 (29.4)
Most (>75%) by privileged clinicians	5/17 (29.4)
Nearly 100% by privileged clinicians	2/17 (11.8)
Other	2/17 (11.8)

Abbreviations: OBGYN, obstetrics-gynecology; POCUS, point-of-care ultrasonography.

^a^ Participants were able to mark several options; only those that were marked first in a participant’s ranking are listed.

### Key Informant Interview Findings

The Figure depicts the key themes associated with POCUS policy implementation from qualitative analysis of interviews using the PRISM framework. Table 3 provides exemplar quotations to highlight each of these themes.

**Figure.**
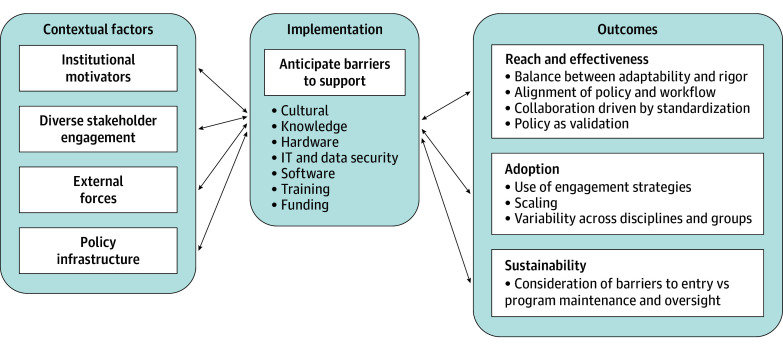
Point-of-Care Ultrasonography Policy Visualized Through the Practical, Robust Implementation and Sustainability Model (PRISM) Framework IT indicates information technology.

**Table 3.** Qualitative Analysis Findings on Development of POCUS Credentialing and Privileging Policy and Implementation

Theme (quotation No.)	Quote
Institutional motivators (1)	“I think as a whole the institution feels like it was the right thing to do all of these steps because now, we feel very confident that medicolegally we’re protected, we’ve got a really well-established algorithm for how to bill...I think that we decreased overall cost of care; I’m ordering less chest x-rays and echo[cardiogram]s than the average hospitalist. I don’t think our length of stay is any longer. I think our outcomes are the same or better, and I think patient satisfaction that I hear at the bedside goes way up.” (Participant 4)
Stakeholder engagement (2)	“Partially because it came from top-down as far as the mandate for the policy, radiology was already involved and they just shared their workflows and [we] didn’t have to recruit IT necessarily, because it was a top-down process.” (Participant 4)
Stakeholder engagement (3)	“But if you can show that this is a priority that’s been identified by the residency program, the residency council, the medical students, and have that bottom-up approach, that can be a huge leverage point for support as well.” (Participant 14)
External forces (4)	“What drove the policy was that our institution entered a partnership with [device] in 2019, with the goal of expanding POCUS to our regional health network...And so through that partnership we recognized a big gap in compliance and credentialing policy when it comes to POCUS.” (Participant 20)
Policy infrastructure (5)	“We wanted to make something that was as flexible as possible and we wanted to make sure that ultrasound leaders for their respective department had as much control over determining who was going to get privileges as possible.” (Participant 7)
Implementation (6)	“The whole point of it taking this long was to create that structure that created sustainability...if there’s feedback from the physicians about this process of rolling this out, it was ‘this is taking too long,’ and the reason it was taking too long was because I wanted to make it sustainable...I would say take your time and get as many stakeholders as you possibly can from different areas to be involved, and it’ll take longer, but I think doing that front end work is going to pay huge dividends in the future, and I think the organization will back you up around that when you have multiple departments and multiple people talking in the same way.” (Participant 2)
Implementation (7)	“I think that just having an overview of the different components of a POCUS program is important at the very get-go, as you’re starting, knowing that these are the things that are needed to be in place for this to be a hundred percent fully functioning program. But I think at the same time, if you were to think that you need all these pieces together and in place for you to start, I don’t think that you’ll ever get off the ground. So being aware of what is important, so that you know what else is missing and then as you build your program, prioritize what we need to do first and then start building these pieces in.” (Participant 6)
Balancing adaptability and rigor (8)	“The worry is [clinicians] surreptitiously use POCUS or that they throw in the towel altogether; neither of which you want. We’ve redesigned our POCUS training a little bit and that’s essentially attached to the policy basically. The idea here is we have a training program that gets you to the point where you can be privileged by this policy, but the policy itself doesn’t specify exactly how we should get there.” (Participant 1)
Aligning policy and workflow (9)	“Just having a lot of machines doesn’t mean people are going to do it. But if you have folks work too hard to find devices, they’re probably not going to. The second piece of that is having a system in place for image management, so that if you’re doing these exams, we track your portfolio and then have a process for providing quality assurance...and so, trying to streamline some of the workflow that allows us to still track their numbers, because I think any extra work you place on somebody makes them less likely to do it.” (Participant 14)
Collaboration driving standardization (10)	“To have a centralized guiding body, ideally by clinicians, a POCUS committee of sorts that decides what should be centralized and what should be decentralized in control and implementation of POCUS and to really get stakeholders up and down the health center involved before you try to jump in. We implemented before that was even a possibility in some ways, and I think now we’re paying for it because it’s hard to walk that process back.” (Participant 1)
Policy as validation (11)	“I think the positive that maybe people don’t realize is that when you have a system in place and you follow all the steps of the system, you essentially are granting yourself the opportunity of getting this done without being questioned. No one can come back and say, ‘Hey, like I don’t actually like what you’re doing, maybe you should stop doing it.’ We have a POCUS policy and we have a privileging policy as well. So yes, we have hospital guidance to do this. Yes, we can do this. We are protected by the umbrella of the system—even though it’s a cumbersome system—it gives you some degree of structure and protection.” (Participant 3)
Use of engagement strategies (12)	“I think clinicians and hospitals really want to adopt POCUS, but they don’t have time...I tried to hit every level that I could. So you’ve got the mandate, you’ve got your infrastructure, you’ve got your champions and then just a lot of encouragement and passion, but you’ve got to really hit those multiple levels; otherwise it’s very difficult to just make any headway, even though people want to adopt.” (Participant 8)
Scaling (13)	“We’re kind of the test market, if you will, because we have GME programs. Other care sites are very deeply invested and eager to see what the experience is here to see if we can expand the privileging, the technology, and the workflow across the health system.” (Participant 5)
Variability across disciplines or groups (14)	“I will say, those groups that are very invested [in policymaking] are all credentialing their providers if they haven’t already been credentialed and getting them up to speed and then have a training process in mind...I do think that there are going to be groups that avoid credentialing because it’s an extra step at this point, and there’s folks that are just completely against it in general.” (Participant 20)
Sustainability (15)	“[Maintenance of privileges] is very hands off as opposed to the initial privileging where they need to look at all the documents, see these are the amount of scans you’re doing, get a letter from someone that already has privileges saying that they’re confident in your scanning technique, review the amount of CME hours, and they review each document...so a much larger barrier to entry, but once entered, an honor system.” (Participant 3)
Sustainability (16)	“We did not want the credentialing guideline to create a barrier for use of POCUS, just to ensure that those who are going to end up using it, teaching it, and billing for it have at least gone through what we hope is the appropriate training...it was important to strike the balance of not making it so restrictive that people are not going to use it, but create a carrot that you can actually reach for as opposed to something that is so high up that no one’s going to use it.” (Participant 15)

Abbreviations: CME, continuing medical education; GME, graduate medical education; IT, information technology; POCUS, point-of-care ultrasonography.

#### Contextual Factors

The context in which a POCUS policy is initiated, developed, implemented, and sustained includes characteristics of both the internal (organization, stakeholders, and clinicians) and external (market- or public-facing) environments as well as features of the policy itself. Four distinct contextual themes emerged as critical to POCUS policy development efforts.

##### Institutional Motivators

In many cases, the decision to initiate standardized privileging policies stemmed from shared values between institutional leaders and POCUS champions. Frequently cited benefits included establishing minimum training standards, increasing revenue, reducing medicolegal risk, supporting educational objectives, adhering to regulatory requirements, and improving patient care (Table 3, exemplar quotation 1).

##### Stakeholder Engagement

Interviewees identified both top-down and bottom-up strategies for stakeholder engagement, noting that different approaches resulted in different outcomes. Top-down approaches supported programs in implementing resource-dependent systems that aid policy oversight and maintenance (eg, equipment, software, and information technology [IT] integration), whereas bottom-up or grassroots approaches were more effective in launching training programs and promoting clinician engagement (Table 3, exemplar quotations 2 and 3). Overall, participants believed that leveraging both strategies simultaneously led to the best results.

##### External Forces

In some institutions, outside forces motivated both institutional leaders and POCUS champions to respond with new institutional policies. These forces included new or expanding partnerships, more accessibility to handheld ultrasonography, payer requirements or incentives, and regulatory requirements (Table 3, exemplar quotation 4). Institutional leaders and POCUS champions collaborated to align the institution with the external forces by developing new policies.

##### Policy Infrastructure

Key elements of participating sites’ policies are summarized in Table 4. Participants noted that effective policy structure and content should reflect organizational capabilities, resources, and priorities. Many policies were designed to include broad, general guidelines at the hospital or health-system level (9 of 19 [47.4%]). A key described feature of these policies is flexibility for departmental or divisional adaptation to specific local needs (Table 3, exemplar quotation 5). This strategy allowed policymakers to set minimum standards across specialties while preserving departmental and divisional autonomy. However, achieving consensus on training standards and POCUS scope across different specialties with variable experiences, resources, and needs was cited to be challenging and time-consuming.

**Table 4.** Institutional Policy Features

POCUS policy features	Institutions, No. (%) (N = 19)
Specified policy users^a^	
Attending physicians	12 (63.2)
Advanced practice clinicians	5 (26.3)
Trainees or noncredentialed users	3 (15.8)
None specified	7 (36.8)
Scope^a^	
Health system	5 (26.3)
Hospital	4 (21.1)
Department	3 (15.8)
Local (eg, division or section)	8 (42.1)
POCUS applications^a^	
Global	10 (52.6)
Specific to examination or organ system	6 (31.6)
Limited vs comprehensive scope	6 (31.6)
Embedded workflow requirements^a^	
Device	1 (5.3)
Image archival	6 (31.6)
Documentation	6 (31.6)
Quality assurance	8 (42.1)
None	9 (47.4)
Initial requirements^b^	
Specific	11 (57.9)
General	8 (42.1)
Maintenance requirements^b^	
Specific	8 (42.1)
General	6 (31.6)
Not specified	5 (26.3)
Internal training or proctoring requirement	
Yes	17 (89.5)
Yes (limited to certain applications)	2 (10.5)
No	0

POCUS, point-of-care ultrasonography.

^a^ Categories are not mutually exclusive.

^b^ “Specific” indicates the policy includes numeric targets for hours of training, number of examinations, and so on; “general” indicates the policy lacks specific targets, defers to an outside organization, and/or uses terms such as *sufficient* or *adequate*.

#### Implementation

Creating a POCUS policy that is endorsed by key stakeholders is a long and multistep process. All participants highlighted the importance of anticipating and addressing barriers proactively, including cultural or institutional resistance to POCUS use, lack of POCUS knowledge and/or local expertise, limited ultrasound machine availability, onerous IT and data security requirements, fragmented documentation and archiving workflows, limited teacher and learner availability, and insufficient funding. Two approaches to overcoming barriers during policy development were described: addressing barriers prior to policy implementation (Table 3, exemplar quotation 6) vs early implementation of the POCUS policy based on current capabilities with plans to iteratively adapt as resources evolved (Table 3, exemplar quotation 7).

#### Reach and Effectiveness

Maximizing impact and participation in POCUS policy are key outcomes of interest for institutions. Four themes emerged in this domain.

##### Balancing Adaptability and Rigor

Participants discussed ideal initial training thresholds for privileging applicants. Approaches aimed to balance training rigor with the desire to reach a broad range of clinicians. Less restrictive policies improved engagement but needed more oversight and maintenance over time, whereas more restrictive policies caused lower initial clinician engagement but less ongoing oversight. Participants also noted that more restrictive thresholds may unintentionally lead to more unprivileged use or discourage POCUS use altogether (Table 3, exemplar quotation 8).

##### Aligning Policy and Workflow

Participants observed that policies and workflows were interrelated, such that more manageable workflows could enable more robust policies and vice versa (Table 3, exemplar quotation 9). For example, centralized image storage and documentation solutions, also called POCUS managers,^1^ streamlined training and QA workflows, which allowed institutions to track POCUS use, offer targeted feedback, and support policy standards. Conversely, fragmented or absent workflows limited policy reach by hindering clinicians’ ability to document initial training requirements, incorporate POCUS into their clinical workflows, and submit images for ongoing QA.

##### Collaboration Driving Standardization

Participants reported that multidisciplinary and multilevel collaborators—including those with budget, financial, IT, and clinical influence—were important for securing resources, addressing initial barriers, and creating sustainability through structured policies and workflows. Institutions that engaged a diverse stakeholder group in these efforts had more sustainable and accessible practices, though often at the expense of time to implementation. For those who pursued early implementation for the sake of timing, having an agreed-upon road map among stakeholders—even if not fully implemented—helped align goals and prioritize key elements based on institutional capabilities. This approach prevented future obstacles and allowed the stakeholders to adapt as resources and institutional support grew (Table 3, exemplar quotation 10).

##### Policy as Validation

Participants described how formal institutional support and systematic implementation strategies for POCUS policies conferred legitimacy to POCUS-practicing clinicians. They also noted that policies could help protect POCUS programs from stressors over time, including institutional leadership changes, financial volatility, and cultural resistance to broader adoption (Table 3, exemplar quotation 11).

#### Adoption

POCUS policies were most effective if appropriately targeted and supported to maximize use and minimize barriers to adoption. We identified 3 emergent themes in this domain.

##### Use of Engagement Strategies

Nearly all participants noted the multilevel challenges of engaging and sustaining clinicians with training and QA practices required for POCUS policies. Often, a small minority of early adopters pursued POCUS credentialing and privileges without significant support. Beyond this group, busy clinicians with competing demands required sufficient incentives, targeted recruitment, and/or local mandates to encourage participation. Consequently, policymakers should be thoughtful about how barriers, real and perceived, may impact privilege adoption (Table 3, exemplar quotation 12).

##### Scaling

Developing and implementing POCUS policies requires time, resources, and content expertise from local and institutional champions. Many institutions effectively leveraged higher-resourced or engaged groups to initiate policy development and then disseminated to the remaining health system (Table 3, exemplar quotation 13). This strategy eased burdens of designing policies de novo and troubleshooting implementation. Notably, policy initiators tended to be associated with trainees (eg, teaching vs nonteaching environments) and clinician POCUS experience (eg, dissemination of EM practices to other specialties) more than institutional type (eg, tertiary vs community-based centers).

##### Variability Across Disciplines and Groups

Policy adoption varied by specialty (Table 3, exemplar quotation 14). Established departments were less likely to adjust existing workflows to fit new policies, while those with limited POCUS integration were more invested in aligning with institutional efforts.

#### Sustainability

Implemented POCUS policies require sustainability infrastructure to support oversight of policy obligations (eg, creation of portfolios, QA, and proctoring) and ongoing programmatic needs (eg, capital, personnel, training, and oversight costs). Different approaches to sustainability included (1) higher barrier to entry (more criteria to gain initial privileges) paired with lower oversight and/or minimal requirements for maintenance of privileges and (2) lower barrier to entry (fewer criteria to gain initial privileges) with increased oversight and higher requirements for maintenance of privileges (eg, ongoing collection and review of images). Many participants commented on difficulty securing adequate funding (eg, for equipment, image archival systems, or other POCUS infrastructure) and protected time (eg, for POCUS champions) to sustain all needed elements of an ideal POCUS policy and developed their policy to reflect their funding capabilities (Table 3, exemplar quotations 15 and 16).

### Policy Content Analysis

A total of 19 POCUS policies were available for content analysis. Table 4 shows POCUS policy features from 19 of 20 participating institutions (95.0%). About half of the policies (9 of 19 [47.4%]) were designed to be broadly applied at the hospital or health-system level, while the others (10 [52.6%]) were specific to department or local (division, section) levels. Most policies (12 [63.2%]) were aimed at attending physicians. About half (10 [52.6%]) conferred general POCUS privileges, though privileging by examination or organ system–specific applications (6 [31.6%]) or by scope of POCUS complexity (limited vs comprehensive; 6 [31.6%]) were also common. Almost half of policies (9 [47.4%]) had no specific workflow requirements, while others outlined practice requirements for image archiving, documentation, and/or QA. For both initial and maintenance credentials and privileges, most policies included specific rather than general requirements, though several policies did not specify any requirements to maintain privileges (5 [26.3%]). Notably, all policies required either internal training or proctoring from POCUS champions within the institution.

## Discussion

This study explored emergent themes in POCUS policy development impacting health systems across the country. To ensure safe and effective POCUS use, POCUS leaders are increasingly turning to policies to standardize training, define competency benchmarks, and establish oversight mechanisms. However, crafting and implementing these policies are challenging due to variable practice patterns and stakeholder opinions, absence of clear guidelines, and the early-stage development of POCUS-related IT systems. Our study used a mixed-methods approach informed by the PRISM framework to understand these complex factors and identify key steps and barriers encountered in policy development and implementation. Despite the presence of significant local context, influences, and barriers to address, we encountered broadly applicable themes that may help institutions in POCUS policy development.

Perhaps most important was the necessity of diverse stakeholder engagement—often achieved by establishing a POCUS committee. These committees included clinicians across multiple specialties, IT and clinical technology personnel, finance administrators, and hospital leaders. They were vital to developing consensus on institutional POCUS policy and identifying and addressing barriers to policy implementation and maintenance of privileges. Discussions with key stakeholders allowed POCUS champions to ensure that policies reflected broad institutional values and were standardized while also allowing for departmental and divisional autonomy. Additionally, approaching financial discussions as an institutionally backed group helped improve credibility and success in garnering needed resources for programmatic oversight and sustainability (eg, software, ultrasound machines, training equipment, and personnel).

A second key lesson was the importance of anticipating and addressing barriers early in POCUS policy development. In interviews, POCUS policymakers espoused 1 of 2 approaches to navigating barriers: a learn-as-you-go approach where policy is introduced early in POCUS program development with frequent iterative adaptation or a retrofit approach where the policy is written to reflect the features of a matured and implemented POCUS program. Each approach has associated pros and cons, and the correct approach was thought to be a function of the local institutional context. Engaging with stakeholders, as noted previously, can help elucidate the best approach for a given institution.

In addition, policymakers should consider available resources and desired outcomes when considering the rigor of the policy around initial privileging and credentialing. There are clear trade-offs to the differing approaches of restrictive policies that require clinicians to meet substantial initial thresholds for use and more permissive policies that encourage use with lower initial thresholds. Restrictive policies may limit uptake and/or unintentionally encourage unsanctioned POCUS use, while permissive policies may lead to sanctioned use that is of lower quality and potentially less safe. To address quality and safety concerns, many institutions that chose restrictive policies were often able to have less rigorous QA programs than the more permissive programs needed. While both types of policies require investment in software and equipment resources, less funding for ongoing oversight and training to support the POCUS policy may be needed in the more restrictive policies. However, limiting use to only a few clinicians may limit the return on investment that an institution sees and may not be consistent with the institutional values that promoted the POCUS policy in the first place. Policymakers need to weigh these outcomes when designing the policy.

Ultimately, the purpose of implementing an institutional POCUS policy is to provide guidance for safer POCUS-informed care, akin to implementing other privileging and credentialing policies. Currently, many health systems are facing rapid unregulated dissemination of POCUS use within the hospital. Factors driving this include increasing availability of affordable handheld devices, a growing body of evidence demonstrating improved quality and patient care outcomes, and limited education in most medical training programs. Designing a policy that aligns with stakeholder values and is supported by institutional funding, leaders, and POCUS champions can add structure and efficiency to training and clinical workflows and promote high-quality POCUS in clinical care. By investing in this work, institutions can harness the full benefits of POCUS across the health care system.

### Limitations

There are several limitations to our study. First, all participants were internal medicine–trained hospitalists, even though this was not an inclusion criterion. We selected these participants because they were more likely to operate in a setting where POCUS was not part of standard training and thus would require additional privileging (in contrast to EM or critical care, where POCUS is well established). We noted that 9 of 19 policies (47.4%) were implemented either at the health system or hospital level and that our interview asked participants to focus on conversations with key stakeholders (eg, hospital administrators, risk management, or other clinical specialties), although explicit opinions of POCUS leaders from other clinical specialties were not included, which may limit applicability. We additionally excluded institutions where policies were not in process or implemented. We acknowledge there is a reasonable divergent opinion that POCUS should not be regulated by policy, but we believed that this was outside of the scope of our research question. In addition, our respondents represented large, urban, teaching, and tertiary care centers, which is reflective of the broader national landscape of systems-level POCUS integration as well as more established POCUS programs. Respondents were predominantly male, which may introduce bias and affect trustworthiness of the data due to males’ expectations, experiences, and communication style.

## Conclusions

To our knowledge, this qualitative study presents the first multi-institutional analysis, outside of national society guidelines and position statements, addressing contextual factors that drive POCUS policy implementation and considering diverse outcomes on patients, clinicians, and institutions. We believe these results may be broadly applicable and are timely for institutions considering how to safely implement and sustain POCUS use.

## Notes

Supplement 1. **eAppendix 1.** Survey Instrument **eAppendix 2.** Interview Guide

Supplement 2.
Data Sharing Statement
