# Supplementary material for: Perspectives on Point-of-Care Ultrasonography Credentialing and Privileging: A Qualitative Study
Source: JAMA Netw Open. 2025 Oct 22;8(10):e2538759. doi: 10.1001/jamanetworkopen.2025.38759 (PMC12547612; doi:10.1001/jamanetworkopen.2025.38759)
Supplement: Supplement 1. — eAppendix 1. Survey Instrument eAppendix 2. Interview Guide [file jamanetwopen-e2538759-s001.pdf]

Supplementary Online Content

Conner SM, Anstey JE, Thomas MK, et al. Perspectives on point-of-care ultrasonography credentialing and privileging: a qualitative study. *JAMA Netw Open*. 2025;8(10):e2538759. doi:10.1001/jamanetworkopen.2025.38759

eAppendix 1. Survey Instrument
eAppendix 2. Interview Guide

This supplementary material has been provided by the authors to give readers additional information about their work.

© 2025 Conner SM et al. *JAMA Network Open*.

eAppendix 1. Survey Instrument

POCUS Survey

Start of Block: Default Question Block

Instructions: This survey is designed to help us understand the status of Credentialing & Privileging Policies for Point of Care Ultrasound at your institution. Please choose the responses that best represent your situation. ***It will take approximately five minutes to complete. None of your responses will be linked to your name or other identifiers.***

Q1 What institute are you affiliated with:

○ Institution name: (1) ____

○ Location (2) ____

---

*

Q2 What is your age in years?

____

Q3 What is your gender identity?

○ Male (1)

○ Female (2)

○ Non-binary (3)

○ Prefer to describe (please describe below) (4)
____

○ Prefer not to answer (5)

© 2025 Conner SM et al. *JAMA Network Open*.

*

Q4 In what year did you complete your training, including fellowship(s)?

____

Page Break

© 2025 Conner SM et al. *JAMA Network Open*.

Q5 Do you have a formal POCUS role at your institution?

○ Yes (2)

○ No (1)

Display this question:

If Do you have a formal POCUS role at your institution? = Yes

Q5b If Yes, what is your title:

____

Page Break

© 2025 Conner SM et al. *JAMA Network Open*.

Q6
How much POCUS-related funding do you receive in terms of FTE?

- ◯ No funding (1)
- ◯ 0-0.2 FTE (2)
- ◯ 0.21-0.4 FTE (3)
- ◯ 0.41-0.6 FTE (4)
- ◯ 0.61-0.8 FTE (5)
- ◯ > 0.8 FTE (6)

Q7 How long have you been using POCUS in clinical care?

	0	1	2	3	4	5	6	7	8	9	10
Years ()											

Page Break

© 2025 Conner SM et al. *JAMA Network Open*.

Q8 Which of the following best characterizes your practice setting?

- ○ Tertiary/quaternary referral center (1)
- ○ Community medical center (2)
- ○ Public/Safety net center (3)
- ○ Other (please describe below): (4)

____

Q9
What is the size of your hospital?

- ○ Small (<100 beds) (1)
- ○ Medium (100-499 beds) (2)
- ○ Large (>500 beds) (3)

Page Break

© 2025 Conner SM et al. *JAMA Network Open*.

Q10 Which of the following best characterizes your practice setting?

○ Rural (1)

○ Suburban (2)

○ Urban (3)

Q11 Is your hospital affiliated with a university?

○ Yes (2)

○ No (1)

Q12 Does your hospital have an internal medicine residency program?

○ Yes (1)

○ No (2)

Page Break

© 2025 Conner SM et al. *JAMA Network Open*.

End of Block: Default Question Block

Start of Block: Block 2

Instructions 2
Part 2: POCUS Policies at Your Institution
Instructions: *For the purpose of this survey, please only consider policies and practices related to diagnostic POCUS in your responses, not procedural.*

Q13
What is the current status of POCUS Credentialling and Privileging policies at your institution?

- ○ Have not started to work on this yet (1)
- ○ Have started, but policy not finalized (2)
- ○ Policy finalized but not implemented yet (3)
- ○ Policy finalized and intermittently used (4)
- ○ Policy finalized and broadly used (5)

Page Break

© 2025 Conner SM et al. *JAMA Network Open*.

End of Block: Block 2

Start of Block: Block 6

Q16 If you have a finalized policy, we would like to review it. Please upload it by clicking here:

Q17
Please rank the following external factors according to the role they played in shaping the credentialing policy at your institution:
______ Reimbursement (1)
______ Adherence to society guidelines (2)
______ Evidence-based medicine practices (3)
______ Medico-legal culture or concerns (4)
______ Patient Safety or Quality (5)
______ Other (please describe): (6)

Page Break

© 2025 Conner SM et al. *JAMA Network Open*.

Q18
Please rank the group of clinicians below according to how likely they are to seek POCUS credentials at your institution:
_______ Emergency Medicine Attendings (1)
_______ Hospitalist Attendings (2)
_______ Primary Care Attendings (3)
_______ Advanced Practice Providers' (4)
_______ OB/GYN Attendings (5)
_______ Anesthesiology Attendings (6)
_______ Critical Care Attendings (7)
_______ Pediatrics Attendings (including subspecialists) (8)
_______ Surgery Attendings (including subspecialists) (9)
_______ Medicine Subspecialty Attendings (10)
_______ Other (please describe): (11)

Page Break

© 2025 Conner SM et al. *JAMA Network Open*.

Q19 What is your best estimate of the number of physicians in your hospitalist group?

____

Q20
What proportion of your hospitalist group would you best estimate currently have POCUS privileges?

- Less than 10% (1)
- 10-25% (2)
- 26-50% (3)
- 51-75% (4)
- 76-100% (5)

Q21
How tightly associated is privileging with diagnostic POCUS use at your institution?

- It is not very tight - Most POCUS exams (>75%) are done by non-privileged clinicians (1)
- It is mixed – Roughly 25-75% of POCUS exams are done by non-privileged clinicians (2)
- It is pretty tight – About >75% of POCUS exams are done by privileged clinicians (3)
- It is very tight – Nearly 100% of POCUS exams are done by privileged clinicians (4)
- Other (please describe below): (5)

____

Page Break

Q22
What type of ultrasound machine does your POCUS policy cover?

- ○ Covers cart-based machines only (1)
- ○ Covers handheld machines only (2)
- ○ Covers both cart-based and handheld machines (3)
- ○ Machine type not specified within the POCUS policy (4)

End of Block: Block 6

Start of Block: Block 5

Part 23 **Part 3: POCUS Practices within your Hospital Medicine Group**

Q24
How would you characterize the level of access to POCUS machines for clinical care that hospitalists have at your institution?

- ○ No ultrasounds are readily available for use in clinical care (1)
- ○ Handheld ultrasounds are more readily available than cart-based ultrasounds (2)
- ○ Cart-based ultrasounds are more readily available than handheld ultrasounds (3)
- ○ Both handheld and cart-based ultrasounds are readily available (4)

© 2025 Conner SM et al. *JAMA Network Open*.

Q25
What options exist for your group to document their POCUS findings (Select all that apply)?

- ☐ No documentation (1)
- ☐ Free text within a clinician's note (2)
- ☐ Smartphrase embedded into clinician's note (3)
- ☐ In a report separate from the Electronic Medical Record (e.g 3rd Party Vendor) (4)
- ☐ In a report integrated with the Electronic Medical Record (e.g. using POCUS specific template within EMR) (5)
- ☐ Using a 3rd party vendor worksheet that integrates into EMR (6)
- ☐ Other (please describe below): (7) ____

Page Break

© 2025 Conner SM et al. *JAMA Network Open*.

Q26
How is your group most commonly archiving POCUS images (Select all that apply)?

- ☐ Not archiving images (1)
- ☐ Onto separate hard drive (Eg. save to USB then transfer to hard drive) (2)
- ☐ Into separate standalone archive (E.g. 3rd party vendor or cloud-based archive) (3)
- ☐ Into Vendor Neutral Archive (VNA) or Picture Archival and Communication System (PACS) via order-based workflow (4)
- ☐ Into VNA or PACS via encounter-based workflow (5)
- ☐ Other (please describe below): (6)
____

Q27 When thinking about all POCUS-users at your institution and their workflow to document and archive POCUS studies, which scenario best describes your institution?

- ○ All POCUS-users utilize a standardized workflow (1)
- ○ There are multiple different POCUS workflows across the institution (2)
- ○ Only a few groups (or departments) utilize a POCUS workflow (3)
- ○ There are no POCUS workflows in use (4)
- ○ Not sure (5)
- ○ Other (please describe below): (6)
____

Page Break

© 2025 Conner SM et al. *JAMA Network Open*.

Q28
Does your group or hospital have a written policy for educational POCUS scans (not used for clinical decision making) scanning?

- ◯ Yes (1)
- ◯ No (2)
- ◯ Unsure (3)

Display this question:

If Does your group or hospital have a written policy for educational POCUS scans (not used for clini... = Yes

Q28b Please upload the written policy

Page Break

© 2025 Conner SM et al. *JAMA Network Open*.

Q29 Which of the following best describes your group's approaches **Quality Assurance (QA) for POCUS images?**

	No standard process exists (1)	No QA is Used (2)	<10% of POCUS images undergo QA (3)	10% -50% of POCUS images undergo QA (4)	>50% of POCUS images undergo QA (5)
Educational only scans (1)	○	○	○	○	○
Scans for clinical practice (2)	○	○	○	○	○
Privileged POCUS users (3)	○	○	○	○	○
Non-privileged POCUS users (4)	○	○	○	○	○

Q30
Please choose the statement that best reflects billing practices **in your hospitalist medicine group**.

○ There is no billing for POCUS services (1)

○ We are working on plans to start billing (2)

○ Some POCUS services are billed for (3)

○ Nearly all POCUS services are billed for (4)

○ Other (please describe below): (5)

____

© 2025 Conner SM et al. *JAMA Network Open*.

Q31
Please choose the statement that best reflect billing practices **at your institution**.

○ There is no billing for POCUS services (1)

○ We are working on plans to start billing (2)

○ Some POCUS services are billed for (3)

○ Nearly all POCUS services are billed for (4)

○ Other (please describe below): (5)
____

End Message
Please proceed to the next page to submit your survey

End of Block: Block 5

Start of Block: Block 4

Q15
When do you think your policy will be finalized?

○ Within 3 months (1)

○ Between 3 and 6 months (2)

○ Between 6 and 9 months (3)

○ Greater than 9 months (please explain below) (4)
____

○ Other (please describe below) (5)
____

End of Block: Block 4

Start of Block: Block 3

© 2025 Conner SM et al. *JAMA Network Open*.

Q14 What do you think are the reasons you have not started working on a POCUS Credentialing & Privileging policy yet (Select all that apply)?

- ☐ Lack of funding (1)
- ☐ Lack of interest (2)
- ☐ Lack of time (3)
- ☐ Lack of clear guidance (4)
- ☐ Medicolegal concerns (5)
- ☐ Other (please describe below): (6) ____

End of Block: Block 3

© 2025 Conner SM et al. *JAMA Network Open*.

eAppendix 2. Interview Guide

Point of Care Ultrasound Credentialing & Policy Practices
Interview Guide **for those with Finalized Policies**

Interviewer: The purpose of this interview is to explore your experiences with creating and maintaining POCUS policies, privileging, and programming at your institution. We will be conducting interviews with several institutions across the U.S. to understand the current landscape of POCUS in diverse clinical settings. There are no right or wrong answers to the interview questions. Your insights about this important topic are invaluable and we are grateful for your time.

This interview is being audio recorded and will be transcribed for analyses. Your name will never be linked to your responses. Data captured as part of this project may appear in publications and/or presentations.

There are two parts to this interview. The first is on Credentialing & Privileging Policies, and the second is on Practice Patterns (e.g., Individuals, Machines, Archiving, Documentation, and QA). Please don't hesitate to ask any questions if you need clarification.

Reminders for interviewers (don't read aloud):

1. Make sure recording and transcription are **both turned on** before starting questions below.
2. Remember that you can probe further on something even if no probe is offered in the guide below ("tell me more about ____" or "can you share more about ____?")
3. Intermittently summarize what you heard the participant say in response to a question to clarify their response

Part 1: Credentialing & Privileging Policies

Interviewer:

We know from your survey that you have a POCUS Credentialing & Privileging policy in place. Can you describe the process you used to develop it (e.g., who was involved, how long did it take)?

1. What factors motivated the creation of the policy at an institutional level (e.g., reimbursement, adherence to society guidelines or evidence-based practice, patient safety, medico-legal culture)?

2. Were there any overarching values that were key points of discussion (e.g., encourage use, patient safety, billing) for the group creating the privileging policy?

3. What, if any, challenges did you face when developing the policy?

   Probe: Are there any medico-legal concerns that your hospital or clinical leadership have about POCUS?

4. Tailored questions related to the specific policy they have (develop after review)

© 2025 Conner SM et al. *JAMA Network Open*.

E.g., Who does this policy apply to – only physicians, or APPs and trainees as well? How did you decide on who to include?

© 2025 Conner SM et al. *JAMA Network Open*.

How does the policy address maintenance of privileges?

How is your institution overseeing the policy, and whose responsibility is it to enforce the standards of the policy?

5. Has your policy been implemented? If yes, tell us about any implementation strategies you used to encourage clinicians to apply for POCUS privileges?

6. What challenges have you encountered implementing your institution's credentialing policy?

   Probe: Have there been concerns about the privileges impacting POCUS use or adoption? How did your group approach these concerns?

7. How does your institution think about the "return on investment" from developing these policies – financial, academic, patient safety, other?

8. Has there been any feedback about POCUS privileges at your institution, either positively or constructively?

Part 2: POCUS Practice Patterns

Interviewer: Thank you for those insights. We're going to shift to questions about practice patterns at your institution. We're especially interested in what facilitators and challenges you've experienced in setting up your current practices related to image archival, documentation, quality oversight, and billing.

1. Describe the process of engaging relevant stakeholders (e.g., clinical care committees, hospital leadership, IT) to implement POCUS workflows.

2. What kind of resources were necessary in the initial implementation of your current POCUS workflows? What was provided? What was lacking? (e.g. IT support, faculty FTE, software, etc.)

3. What factors influenced the practices you use for image archival, documentation, and QA (e.g., reimbursement/billing, evidence-based information, society guidelines)?

4. Describe your plan for sustainability of POCUS workflows at your institution (e.g. funding, regular stakeholder engagement, training, etc).
   Probe: Can you describe your institution's approach to funding support for POCUS (e.g., educators, super-users, QA efforts, machines?

5. In your opinion, are the current POCUS workflows for your group ideal, both in theory and as currently practiced? If not, what barriers have you faced in achieving the ideal workflow?

6. Tailored questions based on survey answers to practice patterns:

© 2025 Conner SM et al. *JAMA Network Open*.

Can you describe your institution's Quality Assurance or active oversight of image archival and documentation?

(If answered yes to billing) How is billing going within your department and your institution for POCUS services? Have there been any challenges?

Do POCUS policies or workflows differ from cart-based machines to handhelds?

7. Does your institution utilize any specific policies or guardrails for educational scanning? Please describe.
   Probe: do you have thoughts on what the ideal process would be for educational scanning?
   Probe: If you have had discussions about educational scanning, who was involved and what concerns were raised?
   Probe: Is there a formal process for notifying the primary team and/or the patient of any findings of the exam which were unexpected or incidental on educational scans?

8. Last question: Now that you know what you know, what advice would you give to other institutions that are just starting this process?

9. (*if time)* Is there anything you'd like to share that we haven't discussed already?

© 2025 Conner SM et al. *JAMA Network Open*.

Point of Care Ultrasound Credentialing & Policy Practices
Interview Guide **for those without Finalized Policies**

Interviewer: The purpose of this interview is to explore your experiences with creating POCUS policies, privileging, and programming at your institution. We will be conducting interviews with several institutions across the U.S. to understand the current landscape of POCUS in diverse clinical settings. There are no right or wrong answers to the interview questions. Your insights about this important topic are invaluable and we are grateful for your time.

This interview is being audio recorded and will be transcribed for analyses. Your name will never be linked to your responses. Data captured as part of this project may appear in publications and/or presentations.

There are two parts to this interview. The first is on Credentialing & Privileging Policies, and the second is on Practice Patterns (e.g., Individuals, Machines, Archiving, Documentation, and QA). Please don't hesitate to ask any questions if you need clarification.

Part 1: Credentialing & Privileging Policies

Interviewer:

1. We know from your survey that you do not yet have a finalized POCUS Credentialing & Privileging policy in place. Can you describe where you currently are in this process?

2. What factors, if any, are motivating you to create this policy (e.g., reimbursement, adherence to society guidelines or evidence-based practice, medico-legal culture)?

3. Have there been any overarching values that are key points of discussion (eg. encourage use, patient safety, billing) for the group creating the privileging policy?

4. What, if any, challenges have you been facing with policy development?
Probe: Are there any medico-legal concerns that your hospital or clinical leadership have about POCUS?

5. Who will your policy apply to? (e.g., only physicians, or APPs and trainees as well?
Probe: How did or will you decide on who to include?

6. How do you think your policy will address maintenance of privileges?

7. How do you think your institution will oversee the policy, and whose responsibility is it to enforce the standards of the policy?

© 2025 Conner SM et al. *JAMA Network Open*.

8. Have you considered how you will implement this policy? In particular, any implementation strategies you plan to use to encourage clinicians to apply for POCUS privileges?

9. What challenges do you think you will have implementing your institution's policy? Probe: Have there been concerns about the privileges impacting POCUS use or adoption? How are you approaching these concerns?

(interviewer start Part 2: practice patterns)

© 2025 Conner SM et al. *JAMA Network Open*.
