# Supplementary material for: Perspectives on Point-of-Care Ultrasonography Credentialing and Privileging: A Qualitative Study
Source: JAMA Netw Open. 2025 Oct 22;8(10):e2538759. doi: 10.1001/jamanetworkopen.2025.38759 (PMC12547612; doi:10.1001/jamanetworkopen.2025.38759)
Supplement: Supplement 2. — Data Sharing Statement [file jamanetwopen-e2538759-s002.pdf]

Data Sharing Statement

Conner. Medical Center POCUS Leaders' Perspectives on Point-of-Care Ultrasonography Credentialing and Privileging. *JAMA Netw Open*. Published October 22, 2025. doi:10.1001/jamanetworkopen.2025.38759

Data
Data available: Yes
Data types: Deidentified participant data, Data (not involving human participants), Data dictionary
How to access data: smc2337@cumc.columbia.edu
When available: With publication

Supporting Documents
Document types: Statistical/analytic code
How to access documents: smc2337@cumc.columbia.edu
When available: With publication

Additional Information
Who can access the data: researchers whose proposed use of the data has been approved
Types of analyses: for a specified purpose
Mechanisms of data availability: with a signed data use agreement
